# Osteoblastic differentiation and P-glycoprotein multidrug resistance in a murine osteosarcoma model

**DOI:** 10.1054/bjoc.1999.1099

**Published:** 2000-03-06

**Authors:** H Takeshita, K Kusuzaki, H Murata, T Suginoshita, M Hirata, S Hashiguchi, T Ashihara, M C Gebhardt, H J Mankin, Y Hirasawa

**Affiliations:** Departments of Orthopaedic Surgery, Pathology I, Kyoto Prefectural University of Medicine, Kyoto, 602, Japan; Department of Orthopaedic Surgery, Otsu Municipal Hospital, 2 Chome, Motomiya, Otsu, Shiga Prefecture, 520, Japan; Department of Orthopaedic Surgery, Massachusetts General Hospital and Harvard Medical School, Boston, MA, 02114, USA

**Keywords:** P-glycoprotein, multidrug resistance, differentiation, malignancy, osteosarcoma

## Abstract

A recent study of multidrug resistance (MDR) 1 gene transfected osteosarcoma cells found a cause-effect relationship between increased expression of P-glycoprotein (P-gp) and a low aggressive phenotype. However, several experimental and clinical studies have observed contradictory findings in that P-gp expression has been associated with tumour progression. In the present study, we characterized P-gp-positive and P-gp-negative single-cell clones of a murine osteosarcoma, to further investigate the relationship between P-gp expression and changes in cell phenotype. Although these clones were all selected by doxorubicin (DOX) exposure, they were heterogeneous with respect to *MDR1* gene expression. The P-gp-positive clones revealed MDR phenotype, whereas the P-gp-negative clones showed no resistance to drugs. Morphological and functional analysis showed that both the P-gp-positive and P-gp-negative clones were more differentiated than the parent cells in terms of enhanced activity of cellular alkaline phosphatase, an increase in well-organized actin stress fibres and enhanced osteogenic activity. Moreover, these subclones all displayed a decrease in malignant potential such as oncogenic activity, tumour growth rate and metastatic ability, regardless of their P-gp status. These results indicate that the observed osteoblastic differentiation and less aggressive phenotype in DOX-selected osteosarcoma cells may not only be explained by the direct effect of P-gp, and accordingly, consideration of the effect of DOX, as well as P-gp, appears to be important. © 2000 Cancer Research Campaign


*British Journal of Cancer* (2000) **82**(7), 1327–1331
© 2000 Cancer Research Campaign
DOI: 10.1054/ bjoc.1999.1099, available online at http://www.idealibrary.com on IDEAL®

### Osteoblastic differentiation and P-glycoprotein multidrug resistance in a murine osteosarcoma model

**H Takeshita[1,3], K Kusuzaki[1], H Murata[1], T Suginoshita[1], M Hirata[1], S Hashiguchi[1], T Ashihara[2], MC Gebhardt[4], HJ Mankin[4] and Y Hirasawa[1]**

Departments of [1]Orthopaedic Surgery and [2]Pathology I, Kyoto Prefectural University of Medicine, Kyoto 602, Japan; [3]Department of Orthopaedic Surgery, Otsu Municipal Hospital, 2 Chome, Motomiya, Otsu, Shiga Prefecture 520, Japan; [4]Department of Orthopaedic Surgery, Massachusetts General Hospital and Harvard Medical School, Boston, MA 02114, USA

**Summary** A recent study of multidrug resistance (MDR) 1 gene transfected osteosarcoma cells found a cause-effect relationship between increased expression of P-glycoprotein (P-gp) and a low aggressive phenotype. However, several experimental and clinical studies have observed contradictory findings in that P-gp expression has been associated with tumour progression. In the present study, we characterized P-gp-positive and P-gp-negative single-cell clones of a murine osteosarcoma, to further investigate the relationship between P-gp expression and changes in cell phenotype. Although these clones were all selected by doxorubicin (DOX) exposure, they were heterogeneous with respect to *MDR1* gene expression. The P-gp-positive clones revealed MDR phenotype, whereas the P-gp-negative clones showed no resistance to drugs. Morphological and functional analysis showed that both the P-gp-positive and P-gp-negative clones were more differentiated than the parent cells in terms of enhanced activity of cellular alkaline phosphatase, an increase in well-organized actin stress fibres and enhanced osteogenic activity. Moreover, these subclones all displayed a decrease in malignant potential such as oncogenic activity, tumour growth rate and metastatic ability, regardless of their P-gp status. These results indicate that the observed osteoblastic differentiation and less aggressive phenotype in DOX-selected osteosarcoma cells may not only be explained by the direct effect of P-gp, and accordingly, consideration of the effect of DOX, as well as P-gp, appears to be important. © 2000 Cancer Research Campaign

**Keywords**: P-glycoprotein; multidrug resistance; differentiation; malignancy; osteosarcoma

P-glycoprotein (P-gp) is a 170 kDa membrane glycoprotein which exports a number of structurally and functionally unrelated drugs from the cell, decreases intracellular accumulation of these drugs, and thus, causes the phenomenon of multidrug resistance (MDR) (Bosch and Croop, 1996). P-gp has been detected in a variety of human cancers, including osteosarcoma, and may be at least partially responsible for the observed drug resistance in some current chemotherapy regimens (Chan et al, 1991; Balidini et al, 1995).

There has been recent interest in changes in tumour cell phenotype in relation to P-gp expression. For example, studies on human neuroblastoma and mouse sarcoma cell lines have shown that increased expression of P-gp correlates with a decrease in oncogenic activity and expression of a more differentiated phenotype (Bielder, 1994; Bielder and Spengler, 1994). Similar results have been obtained in a variety of MDR cell lines. Furthermore, a transfection study of *MDR1* gene in osteosarcoma cells clearly demonstrated a cause-effect relationship between P-gp expression and a less aggressive phenotype (Scotlandi et al, 1999). In contrast, studies on rat liver carcinogenesis have consistently indicated an association of P-gp overexpression with tumour progression (Bradley et al, 1992; Bradley and Ling, 1994). In an immunohistochemical analysis of human colon cancer, P-gp was overexpressed in relation to the increased invasiveness of tumour cells (Weinstein et al, 1991). It thus appears that although P-gp may play a role in reduced malignancy, observed phenotypic changes do not always reflect the function of P-gp.

We recently isolated several clones of a murine osteosarcoma by pulse exposures of the parental cells to doxorubicin (DOX) followed by single-cell culture (Takeshita et al, 1996). Many of these single-cell clones overexpressed P-gp, but some clones did not express detectable P-gp. The P-gp-positive clones displayed a relatively low level of resistance similar to clinically observed drug resistance, and retained tumorigenic activity, and thus, appeared to be a useful model for additional investigations into MDR osteosarcoma (Takeshita et al, 1998). In the present study, we further characterized these P-gp-positive and P-gp-negative osteosarcoma clones, and found that DOX-selected osteosarcoma cells express osteoblastic differentiation and a low aggressive phenotype, regardless of their P-gp status.

### MATERIALS AND METHODS

### Cell lines

Two P-gp-positive (MOS/ADR1 and MOS/ADR2) and two P-gp-negative (MOS/RV1 and MOS/RV2) single-cell clones as well as the parent osteosarcoma cell line were used for the present study. These clones were selected by exposure to DOX, as described previously (Takeshita et al, 1996). The MOS/ADR1 and MOS/RV1 cells consisted of non-polyploid cells having DNA

*Received 7 May 1999*
*Revised 14 September 1999*
*Accepted 9 October 1999*

*Correspondence to:* H Takeshita

**Table 1** Pgp expression, DNA content and MDR phenotype

| Cell line | P-gp[a] | DI | $IC_{50}$ (μg ml$^{-1}$) DOX | VCR | ETP | MTX |
|---|---|---|---|---|---|---|
| MOS | –[b] | 1.4 | 0.022 (1)[d] | 0.0049 (1) | 0.065 (1) | 0.022 (1) |
| MOS/RV1 | – | 1.3 | 0.011 (0.5) | 0.0023 (0.46) | 0.061 (0.93) | 0.018 (0.81) |
| MOS/RV2 | – | 2.5 | 0.035 (1.5) | 0.0023 (0.46) | 0.095 (1.4) | 0.010 (0.45) |
| MOS/ADR1 | +[c] | 1.3 | 0.16 (7.2) | 0.074 (15) | 0.28 (4.3) | 0.017 (0.77) |
| MOS/ADR1 | + | 2.4 | 0.41 (18) | 0.060 (12) | 0.85 (13) | 0.025 (1.1) |

[a]P-gp, P-glycoprotein; DI, DNA index; DOX, doxorubicin; VCR, vincristine; ETP, etoposide; MTX, methotrexate. [b]–, Pgp was not stained positively with mAb C219. [c]+, More than 50% of cells were stained positively with mAb C219. [d]Numbers in parentheses represent relative values as compared with the $IC_{50}$ for the parent MOS cells.

indices similar to that of the parent cells, and the other two clones consisted of polyploid cells. The cell lines were maintained at 37°C in a humidified incubator containing 5% carbon dioxide in Dulbecco's modified Eagle medium supplemented with 15 mmol l$^{-1}$ HEPES buffer, 10% fetal calf serum and an antibiotic solution.

### P-gp expression and cellular DNA content

Cells grown on coverslips were washed with phosphate-buffered saline (PBS), fixed with acetone for 30 min, and stained by the indirect immunofluorescence method. The primary monoclonal antibody C219 (Centcor Diagnostics, USA) was applied for 20 h at 4°C (5 μg ml$^{-1}$). After washing with PBS, the cells were incubated with fluorescein isothiocyanate (FITC)-conjugated F (ab)´2 goat anti-mouse IgG (Caltag Laboratories, USA) for 1 h and rewashed with PBS. P-gp immunofluorescence was examined with a fluorescence microscope. For the DNA content analysis, cells were harvested by trypsinization, smeared onto glass slides with a cyto-tec centrifuge, fixed with 70% ethanol, treated with 0.1% RNAase and then stained with propidium iodide. The cellular DNA content was measured by cytofluorometry.

### MDR phenotype

MDR phenotype of the cell lines was determined using the MTT (3-(4,5-dimethylthiazal-2-yl)-2,5-diphenyltetrazolium bromide) assay described by Hansen et al (1989). $IC_{50}$ values (concentrations of the drugs that produce 50% growth inhibition) were determined for each of the cell lines.

### Cellular alkaline phosphatase (ALP) activity

Cells were harvested by trypsinization, suspended in 10 mmol 1$^{-1}$ Tris–hydrochloride (pH 7.4) containing 1 mmol 1$^{-1}$ magnesium chloride, 20 mmol l$^{-1}$ zinc chloride, 0.02% (weight per volume) sodium azide and 0.1% Triton X-100, and sonicated for 30 s. The sonicates were centrifuged for 15 min at 8000 ***g*** at 4°C. ALP activity was determined by measuring spectrophotometrically (410 nm at 37°C) the release of p-nitrophenol from p-nitrophenylphosphatase using the method of Lowry (1955). Protein was measured using a protein assay kit (BCA; Pierce, USA).

### Fluorescent staining of cellular actin

Cells grown on coverslips were rinsed briefly in PBS, fixed for 5 min with buffered formalin and then washed with PBS. The cells were then permeabilized by a 2-min exposure to 0.1% Triton X-100 in PBS at room temperature, followed by rinsing with PBS. FITC-conjugated phalloidin (Sigma; Wulf et al, 1979) was applied to the cells at a concentration of 0.05 mg ml$^{-1}$ in PBS, and was allowed to react for 40 min in a humid atmosphere. After washing with PBS, the cells were examined by a fluorescence microscope.

### In vivo tumour growth

After the cells were trypsinized, $1 \times 10^7$ cells in a small volume of medium was injected subcutaneously (s.c.) in C3H/Sed mice. The tumours were measured weekly in two perpendicular dimensions until their volumes exceeded 2000 mm$^3$. The tumour volumes were estimated using the formula $(a^2 \times b)/2$, where a is the shorter and b is the longer of the two measurements. The primary tumours in s.c. and both lungs were then resected. One half of each tumour was cultured for the study of P-gp staining. The other half of the tumour and lungs were fixed with buffered formalin, and then processed for histological examination. Lung metastases of the tumour cells were assessed using both macroscopic and microscopic findings of the lung specimens.

### RESULTS

### P-gp expression and MDR phenotype in clonal cell lines

Compared with the parent cell line, the P-gp-positive lines, MOS/ADR1 and MOS/ADR2, were more resistant to DOX, vincristine and etoposide, all of which are known to be substrates for P-gp (Table 1). In contrast, the P-gp-negative lines, MOS/RV1 and MOS/RV2 were not resistant to these drugs. All cell lines displayed similar sensitivity to methotrexate, which is not a substrate for P-gp. There was no relationship between P-gp expression and cellular polyploidization. Considering that these subclones were all isolated from the surviving cells after exposure of the parent cells to DOX, the P-gp-negative single cell clones appeared to be composed of revertants of P-gp-positive cells. Indeed, we confirmed that P-gp-mediated drug-resistant phenotype was rapidly lost in DOX-free medium, using a DOX accumulation assay for colony-forming cells (data not shown).

### Differentiation of the DOX-treated osteosarcoma clones

Both the P-gp-positive and P-gp-negative clones demonstrated higher ALP activity than the parent cell line (Figure 1).

 © 2000 Cancer Research Campaign

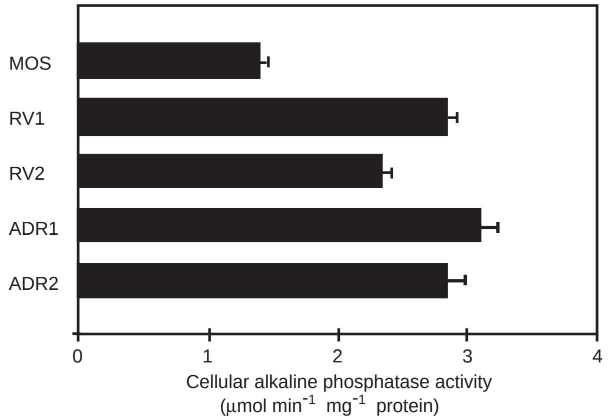

**Figure 1** Cellular ALP activity determined by the method of Lowly. The data were representative of two independent experiments with similar results. The results are expressed as µmol $min^{-1}$ $mg^{-1}$ protein. Bars, standard deviation

Morphologically, these clones were characterized by the presence of those cells which had well-organized actin stress fibres in their cytoplasm. In the parent cells, however, the actin fibres were either sparsely distributed or diffusely spread throughout the cytoplasm (Figure 2 A–E). Although all the tumours produced by inoculation of cultured cells into mice s.c. exhibited histological characteristics of osteogenic sarcoma, they varied in cellularity and the amount of bone and osteoid matrix (Figure 2 F–J). The tumours resulting from inoculation of the parent cells consisted of dense tumour cells with a small amount of relatively immature osteoid formation. The tumours produced from inoculation of the MOS/ADR1 cells showed more abundant bone and osteoid formation than those produced by the parent cell line. Similar histological findings were observed in the tumours produced from the MOS/ADR2 cells or the MOS/RV1 cells. The tumours produced by the MOS/RV2 cells revealed relatively higher cellularity.

### In vivo tumour growth and metastatic potential

The parent osteosarcoma cell line (MOS) showed 100% tumorigenicity following a s.c. injection of these cultured cells into mice, all of which developed metastatic lesions in the lungs (Table 2). In mice inoculated with the MOS cells, the tumours grew rapidly with a doubling time of 4.9 days. In contrast, all four subclones, which were isolated after exposure of the parent cells to DOX, demonstrated a decrease in tumorigenicity, growth rate and metastatic potential. No mice inoculated with these subclones developed metastases in the lungs.

### DISCUSSION

Recent investigations have shown that adverse prognosis in P-gp overexpressing osteosarcomas may result simply from resistance to chemotherapy, but not from biological aggressiveness (Scotlandi et al, 1996). In fact, studies of *MDR1* gene-transfected osteosarcoma cells demonstrated that P-gp may have a direct or at least an indirect role in a decrease in aggressive tumour phenotype (Scotlandi et al, 1999). In the present study, we characterized the P-gp-positive and P-gp-negative single-cell clones of a murine osteosarcoma, to further investigate the relationship between P-gp expression and the observed changes in cell phenotype.

Our results demonstrated that the P-gp-positive MDR clones and P-gp-negative drug-sensitive clones both expressed more differentiated and less aggressive tumour phenotype, when compared to the parent osteosarcoma cells. These clones exhibited enhanced activity of cellular alkaline phosphatase, an increase in well-organized actin stress fibres and enhanced osteogenic activity. Moreover, they all displayed a decrease in oncogenic activity, tumour growth rate and metastatic ability, regardless of their P-gp status. Regarding these observations, two different explanations can be discussed.

Because the osteosarcoma subclones described here were all isolated from the surviving cells following DOX exposure, the P-gp-negative, drug-sensitive clones appeared to be composed of revertants of P-gp overexpressing cells. Based on this hypothesis, it is possible that the reduced malignancy found in the P-gp-positive and P-gp-negative clones may be due to an indirect effect of P-gp. In this case, as pointed out by Scotlandi, expression of the *MDR1* gene may result in coactivation of other genes responsible for the decreased malignancy, and then, only the *MDR1* gene expression was partially suppressed in the P-gp-negative clones.

Another possible explanation may be that DOX produced a significant effect on osteoblastic differentiation and a decrease in aggressive phenotype both in the P-gp-positive and P-gp-negative clones, although P-gp also played a role in reduced malignancy in the P-gp-positive clones. Unlike *MDR1* gene transfectants (Scotlandi et al, 1999), our panel of osteosarcoma subclones selected by DOX showed morphological and functional changes in their cellular differentiative state. Moreover, DOX is known to promote cellular differentiation (Morceau et al, 1996). Thus, consideration of the effect of DOX, as well as P-gp, appeared to be more relevant to explain our experimental results.

Although our results were obtained from only one model of MDR osteosarcoma, they might help to explain the divergent findings regarding the relationship between P-gp expression and malignancy. It is known that *MDR1* gene expression can be

**Table 2** doubling time and metastatic ability[a]

| Cell line | *n* | Tumour (%) | Doubling time (day) | Metastases (%) |
|---|---|---|---|---|
| MOS | 12 | 12/12 (100) | 4.9 | 12/12 (100) |
| MOS/RV1 | 12 | 3/12 (25) | 15.0 | 0/12 (0) |
| MOS/RV2 | 12 | 8/12 (66) | 7.9 | 0/12 (0) |
| MOS/ADR1 | 12 | 7/12 (58) | 10.0 | 0/12 (0) |
| MOS/ADR2 | 12 | 6/12 (50) | 11.8 | 0/12 (0) |

[a]Tumorigenicity and metastatic ability are shown as a total of two independent experiments with similar results. In each experiment, cells ($1 \times 10^7$) were injected into six mice, and tumour growth was monitored until 3 months after injection. Doubling times represent means of two to six determinations in the experiment 2

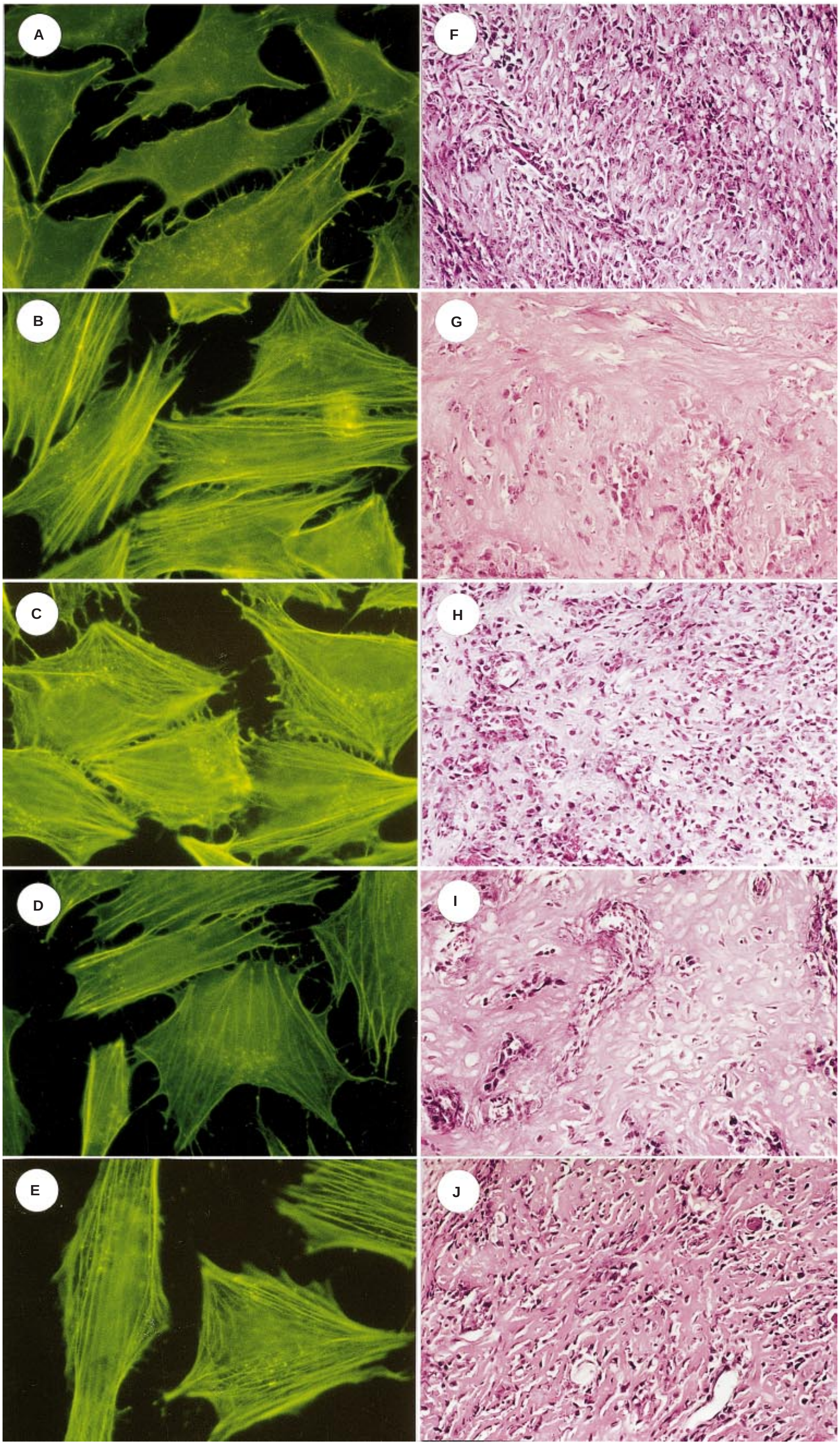

**Figure 2** Fluorescent staining of cellular actin by FITC-conjugated phalloidin (**A**–**E**) (magnification × 1000), and haematoxylin and eosin staining of histological sections of tumours in mice (**F**–**J**) (magnification × 40). **A** and **F**, parent cell line. **B** and **G**, MOS/RV1. **C** and **H**, MOS/RV2. **D** and **I**, MOS/ADR1. **E** and **J**, MOS/ADR2

*© 2000 Cancer Research Campaign*

stimulated by a broad spectrum of structurally and functionally unrelated agents, including anticancer drugs (Fardel et al, 1997), differentiation-inducing agents (Mickerley et al, 1989), growth factors (Hirsch-Ernst et al, 1995), carcinogens (Fairchild et al, 1987), tumour necrosis factor alpha (Hirsch-Ernst et al, 1998), and UV-irradiations (Uchiumi at al, 1993). All these agents may be able to cause P-gp overproduction as well as their known effects in a cell. In addition, P-gp itself is also likely to have positive effects on a decrease in aggressive phenotype. Therefore, the combined effects of all these factors should be taken into consideration. For example, the use of an anticancer drug for selection of MDR cells is expected to result in coexpression of P-gp and the reduced malignant phenotype, because the drug and P-gp may both suppress aggressive phenotype. In contrast, when *MDR1* gene expression is stimulated by carcinogens or by growth factors, P-gp expression may be associated with either more aggressive or less aggressive tumour phenotype depending on the total effects of these factors and P-gp. It is also possible that several factors having different actions may simultaneously stimulate *MDR1* gene expression. Moreover, cell phenotype expression might be affected by the tissue origin of the cell. To prove these hypotheses, however, further studies using various stimulators of *MDR1* gene expression as well as different types of cells are needed.

In conclusion, we demonstrated that both the P-gp-positive and P-gp-negative osteosarcoma clones selected by DOX exposure expressed osteoblastic differentiation and less aggressive tumour phenotype. It is known from clinical studies that human osteosarcoma cells often appear to exhibit histological differentiation after drug treatment (Jaffe, 1993; Link, 1993), and to express a high level of P-gp in the relapsed tumours (Serra et al, 1995). Studies are underway to determine the relationship between P-gp-mediated MDR and differentiation in a clinical setting.

### ACKNOWLEDGEMENT

This work was supported in part by a Grant-in-Aid from the Ministry of Education, Science and Culture of Japan.

### REFERENCES

Baldini N, Scotlandi K, Barbanti-Brodano G, Manara MC, Maurici D, Bacci G, Bertoni F, Picci P, Sottili S, Campanacci M and Serra M (1995) P-glycoprotein expression and outcome in high grade osteosarcoma. *N Engl J Med* **333**: 1380–1385

Biedler JL (1994) Drug resistance: genotype versus phenotype. Thirty-second GHA Clowes Memorial Award Lecture. *Cancer Res* **54**: 666–678

Biedler JL and Spengler BA (1994) Reverse transformation of multidrug-resistant cells. *Cancer Metastasis Rev* **13**: 191–207

Bosch I and Croop J (1996) P-glycoprotein multidrug resistance and cancer. *Biochim Biophys Acta* **1288**: F37–F54

Bradley G and Ling V (1994) P-glycoprotein, multidrug resistance and tumor progression. *Cancer Metastasis Rev* **13**: 223–233

Bradley G, Sharma R, Rajalakshmi S and Ling V (1992) P-glycoprotein expression during tumor progression in the rat liver. *Cancer Res* **52**: 5154–5161

Chan HSL, Haddad G, Thorner PS, DeBoer G, Lin UP, Ondrusek N, Yeger H and Ling V (1991) P-glycoprotein expression as a predictor of the outcome of therapy for neuroblastoma. *N Engl J Med* **325**: 1608–1614

Fairchild CG, Ivy SP, Rushmore T, Lee G, Koo P, Goldsmith ME, Myers CE, Farber E and Cowan KH (1987) Carcinogen-induced mdr overexpression is associated with xenobiotic resistance in rat preneoplastic liver nodules and hepatocellular carcinomas. *Proc Natl Acad Sci USA* **84**: 7710–7705

Fardel O, Lecureur V, Daval S, Corlu A and Guillouzo A (1997) Up-regulation of P-glycoprotein expression in rat liver cells by acute doxorubicin treatment. *Eur J Biochem* **246**: 186–192

Hansen MB, Nielsen SE and Berg K (1989) Re-examination and further development of a precise and rapid dye method for measuring cell growth/cell kill. *J Immunol Methods* **119**: 203–210

Hirsch-Ernst KI, Ziemann C, Schmitz-Salue C, Foth H and Kahl GF (1995) Modulation of P-glycoprotein and mdr1b mRNA expression by growth factors in primary rat hepatocyte culture. *Biochem Biophys Res Commun* **215**: 179–185

Hirsch-Ernst KI, Ziemann C, Foth H, Kozian D, Schmitz-Salue C and Kahl GF (1998) Induction of mdr1b mRNA and P-glycoprotein expression by tumor necrosis factor alpha in primary rat hepatocyte cultures. *J Cell Physiol* **176**: 506–515

Jaffe N (1993) Osteosarcoma: contribution of chemotherapy to treatment of the primary tumor. In: *Frontiers of Osteosarcoma Research: Interdisciplinary Survey of Clinical and Research Advances*, Novak JF and Mcmaster JH (eds), pp. 25–40. Hogrefe & Huber: Kirkland

Link MP (1993) Preoperative and adjuvant chemotherapy in osteosarcoma. In: *Frontiers of Osteosarcoma Research: Interdisciplinary Survey of Clinical and Research Advances*, Novak JF and Mcmaster JH (eds), pp. 41–49. Hogrefe & Huber: Kirkland

Lowly OH (1955) Micromethods for the assay of enzyme II. Specific procedures. Alkaline phosphatase. *Meth Enzymol* **4**: 371–372

Mickerley LA, Bates ASE, Richert ND, Currier S, Tanaka S, Foss F, Rosen N and Fojo AT (1989) Modulation of the expression of a multidrug resistance gene (mdr-1/P-glycoprotein) by differentiating agents. *J Biol Chem* **264**: 18031–18040

Morceau F, Chenais B, Gillet R, Jardillier JC, Jeannesson P and Trentesaux C (1996) Transcriptional and post-transcriptional regulation of erythroid gene expression in anthracyclin-induced differentiation of human erythroleukemic cells. *Cell Growth Differ* **7**: 1023–1029

Scotlandi K, Serra MC, Nicoletti G, Vaccari M, Manara MC, Nini G, Landuzzi L, Colacci A, Bacci G, Bertoni F, Picci P, Campanacci M and Baldini N (1996) Multidrug resistance and malignancy in human osteosarcoma. *Cancer Res* **56**: 2434–2439

Scotlandi K, Manara MC, Serra M, Benini S, Maurici D, Caputo A, Giobanni CD, Lollini P-L, Nanni P, Picci P, Campanacci M and Baldini N (1999) The expression of P-glycoprotein is causally related to a less aggressive phenotype in human osteosarcomas. *Oncogene* **18**: 739–746

Serra M, Scotlandi K, Manara MC, Maurici D, Benini S, Sarti M, Campanacci M and Baldini N (1995) Analysis of P-glycoprotein expression in osteosarcoma. *Eur J Cancer* **31A**: 1998–2002

Takeshita H, Gebhardt MC, Springfield DS, Kusuzaki K and Mankin HJ (1996) Experimental models for the study of drug resistance in osteosarcoma; P-glycoprotein-positive murine osteosarcoma cell lines. *J Bone Joint Surg* **78-A**: 366–375

Takeshita H, Kusuzaki K, Tsuji Y, Hirata M, Hashiguchi S, Nakamura S, Murata H, Ashihara T and Hirasawa Y (1998) Avoidance of doxorubicin resistance in osteosarcoma cells using a new quinoline derivative, MS-209. *Anticancer Res* **18**: 739–742

Uchiumi T, Kohno K, Tanimura H, Matsuo K, Sato S, Uchida Y and Kuwano M (1993) Enhanced expression of the human multidrug resistance 1 gene response to UV light irradiation. *Cell Growth Differ* **4**: 147–157

Weinstein RS, Jakate SM, Dominguez JM, Lebovits MD, Koukoulis GK, Kuszak JR, Grogan TM, Saclarides TJ, Roninson IB and Coon JS (1991) Relationship of the expression of the multidrug resistance gene product (P-glycoprotein) in human colon carcinoma to local tumor aggressiveness and lymph node metastasis. *Cancer Res* **51**: 2720–2726

Wulf E, Deboben A, Bautz FA, Faulstich H and Wieland Th (1979) Fluorescent phallotoxin, a tool for the visualization of cellular actin. *Proc Natl Acad Sci USA* **76**: 4498–4502

*© 2000 Cancer Research Campaign* 
